# Animal, Herb, and Microbial Toxins for Structural and Pharmacological Study of Acid-Sensing Ion Channels

**DOI:** 10.3389/fphar.2020.00991

**Published:** 2020-07-08

**Authors:** Dmitry I. Osmakov, Timur A. Khasanov, Yaroslav A. Andreev, Ekaterina N. Lyukmanova, Sergey A. Kozlov

**Affiliations:** ^1^ Shemyakin-Ovchinnikov Institute of Bioorganic Chemistry, Russian Academy of Science, Moscow, Russia; ^2^ Institute of Molecular Medicine, Sechenov First Moscow State Medical University, Moscow, Russia

**Keywords:** acid-sensing ion channels, natural compounds, ligand receptor interaction, structural features, analgesia, drug development

## Abstract

Acid-sensing ion channels (ASICs) are of the most sensitive molecular sensors of extracellular pH change in mammals. Six isoforms of these channels are widely represented in membranes of neuronal and non-neuronal cells, where these molecules are involved in different important regulatory functions, such as synaptic plasticity, learning, memory, and nociception, as well as in various pathological states. Structural and functional studies of both wild-type and mutant ASICs are essential for human care and medicine for the efficient treatment of socially significant diseases and ensure a comfortable standard of life. Ligands of ASICs serve as indispensable tools for these studies. Such bioactive compounds can be synthesized artificially. However, to date, the search for such molecules has been most effective amongst natural sources, such as animal venoms or plants and microbial extracts. In this review, we provide a detailed and comprehensive structural and functional description of natural compounds acting on ASICs, as well as the latest information on structural aspects of their interaction with the channels. Many of the examples provided in the review demonstrate the undoubted fundamental and practical successes of using natural toxins. Without toxins, it would not be possible to obtain data on the mechanisms of ASICs’ functioning, provide detailed study of their pharmacological properties, or assess the contribution of the channels to development of different pathologies. The selectivity to different isoforms and variety in the channel modulation mode allow for the appraisal of prospective candidates for the development of new drugs.

## Introduction

Natural compounds synthesized by marine and terrestrial inhabitants of three kingdoms (animals, plants, and bacteria) can be considered simultaneously a hazard and a remedy for life quality improvements. Since ancient times, pharmacy has been based inherently on the discovery, examination, and implementation of bioactive molecules, mainly from plants, for treatment of humans. However, the appearance of effective separation methods provided a significant impetus to the promotion of natural compounds from other organisms on the drug market, since it became possible to remove highly toxic components of venoms. The modern growth development of genomics, proteomics, and biotechnology make possible successful study of bioactive molecules, even from very rare animals. As a result, there is an overabundance of structural and functional information of natural compounds that was not confirmed by cellular target specificity. In this review, we only include natural ligands capable of interacting with an acid-sensing ion channels (ASICs).

ASICs are Na^+^-selective channels abundantly expressed in neurons of the peripheral and central nervous systems, where they perform an important function in signal transmission associated with a local change in pH. They are of the most sensitive sensors of acidification in the organism. Indirect confirmation of the existence of these channels was first obtained in the early 1980s, when sodium-selective and rapidly activated and desensitized transient current was detected on mammalian sensory neurons in response to a sharp acidification of the extracellular medium (Krishtal and Pidoplichko, 1980; Krishtal, 2015). In the mid-1990s, the channels with such properties were cloned and expressed and then got their modern name of “acid-sensing ion channels” (Waldmann et al., 1997).

Four genes encode six isoforms of ASICs in mammals: ASIC1a, ASIC1b, ASIC2a, ASIC2b, ASIC3, and ASIC4 (Wemmie et al., 2006). In neurons of the peripheral nervous system (PNS), all isoforms and especially ASIC3 have been found, with the exception of ASIC4. In neurons of the central nervous system (CNS), all isoforms (except ASIC1b) have also been detected, and the ASIC1a isoform is predominant (Deval and Lingueglia, 2015; Schuhmacher and Smith, 2016). ASICs can form homo- and heterotrimeric complexes. Thus, heteromeric complexes of all isoforms have been detected in both CNS (ASIC1a/ASIC2a, ASIC1a/ASIC2b, ASIC1a/ASIC4) and PNS neurons (ASIC1a/ASIC1b, ASIC1a/ASIC3, ASIC1b/ASIC3, ASIC2b/ASIC3) (Lingueglia et al., 1997; Alvarez de la Rosa et al., 2002; Askwith et al., 2004; Gautam and Benson, 2013; Wu et al., 2016).

Ligands, controlling the function of ASICs, were found amongst animal polypeptides, microbial metabolites, and in plant extracts pointing on high-relevant evolutionary role of these channels. In this review, we present the widest and most complete list of natural ligands and discuss modern structural aspects and practical applications of these compounds for the study of ASICs physiological and pathological roles in an organism.

## Biophysical Properties of Acid-Sensing Ion Channels

**Figure 1:**
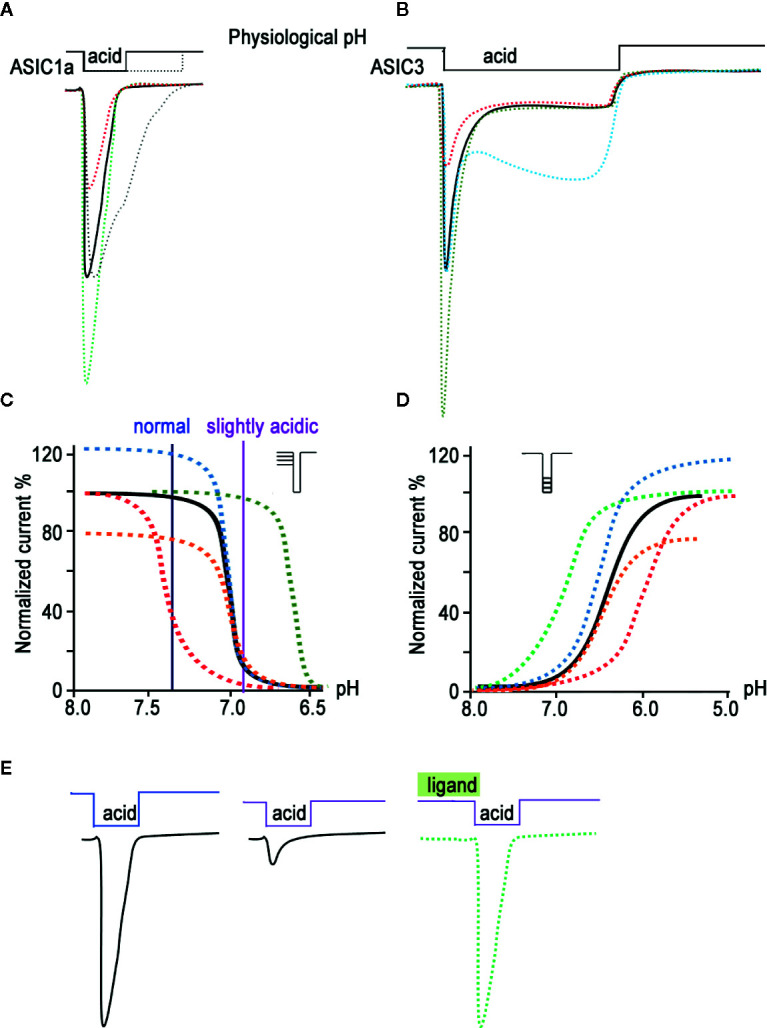
Biophysical properties of ASICs. The characteristic traces of currents recorded in whole-cell configuration are presented for ASIC1a **(A)** and for ASIC3 **(B)** as a black line; currents modified by ligands application are represented in green (potentiation) and red (inhibition). The gray line in **(A)** and cyan line in **(B)** reflect the process of desensitization kinetics change. The pH dependence of the channel gating is shown as a result of steady-state desensitization (SSD) **(C)** and as dependence of currents’ amplitude by various acidic stimuli applied **(D)** for the channel alone (black line). The potentiating effect of ligands is demonstrated as green and blue curves, and the inhibiting effect as red and orange curves. **(E)** Current traces reflect the effect of pH-dependent SSD. In a weakly acidic environmental medium, the channel loses its ability to fully respond to a stronger acid stimulus; however, the addition of a ligand is able to restore its properties (green line).

The subunit composition of each ASIC determines its pH sensitivity, kinetics, and pharmacology (Escoubas et al., 2000; Diochot et al., 2004; Hesselager et al., 2004). Sensitivity to acidic pH varies between different isoforms as follows: ASIC1a and ASIC3 channels are the most sensitive, with a half-activation pH (pH_50_) of about 6.4–6.6; ASIC1b channels occupy an intermediate position (pH_50_ 5.9–6.3); and ASIC2a channels are the least sensitive (pH_50_ 4.3–4.9) (Boscardin et al., 2016). ASIC2b and ASIC4 do not respond to the acid stimulus and apparently only form heteromeric channels with other isoforms, thereby influencing the function of the channel as a whole (Deval et al., 2004; Donier et al., 2008; Sherwood et al., 2011). Under the action of an acid pulse, all functional ASICs form a rapidly activated current, which then desensitizes at different rates (**Figure 1A** for ASIC1a subtype) (Gründer and Pusch, 2015; Osmakov et al., 2019a). According to the kinetics of desensitization, the currents of ASIC3 channels stand apart; in other words, a rapidly desensitizing (transient) component is followed by a non-desensitizing current (sustained component), which lasts as long as the stimulating pulse (**Figure 1B** for ASIC3 subtype) (Salinas et al., 2009; Osmakov et al., 2014). A common property of all subtypes is an increase of current amplitude upon more acid stimulation up to a certain saturation level, so the proton activation curve (ligand receptor dependence) has a characteristic shape and pH value of 50% response (**Figure 1D**).

Another property of ASICs is their ability to reach steady-state desensitization (SSD)—that is, to transition into a desensitized state from a closed state, bypassing the activation process (**Figures 1C, E**). This phenomenon is observed when the proton concentration in the environment increases insignificantly and does not cause activation of the channels but the channels respond to the next strong acid stimulus, either much weaker or non-existent (Waldmann et al., 1997; Alijevic and Kellenberger, 2012).

For compounds isolated from natural sources (plant, microbial, and animal peptides; see below), both positive and negative modulating effects on ASICs have been described. Thus, some ligands show the ability to reduce or, conversely, increase the amplitude of the acid-induced current of one or several ASIC isoforms (**Figures 1C, D** orange and blue curves, respectively). Other ligands are able to increase or decrease the desensitization time constant of this current (“narrowing” or “widening” the current trace, respectively), thereby affecting the kinetics of a channel’s transition from one state to another. Ligands can also shift the dependence of channel activation (**Figure 1D**). On the graph, the ligand’s potentiating effect is expressed as a curve shift towards higher pH values (green curve), while its inhibitory effect is expressed as a shift towards lower pH values (red curve). Another group of ligands does not act on the activation of the channel directly but produces an effect on the SSD of the channel. In this case, the sensitivity of the channels at resting state to protons either increases (the curve shifts towards higher pH values, and the channel becomes poorly susceptible to acid stimuli; **Figure 1C** red curve) or decreases (the curve shifts towards lower pH values; **Figure 1C** green curve). Toxins usually have a mixed effect, for example combining the amplitude change with a shift of activation or SSD. Moreover, toxins often change the kinetics of activation and inactivation processes, leading to alterations in the slope of curves. Thus, a large arsenal of natural ligands for ASICs has been accumulated to this day, and it is possible to control the biophysical properties of these channels differently.

## The Architecture of Acid-Sensing Ion Channels

**Figure 2:**
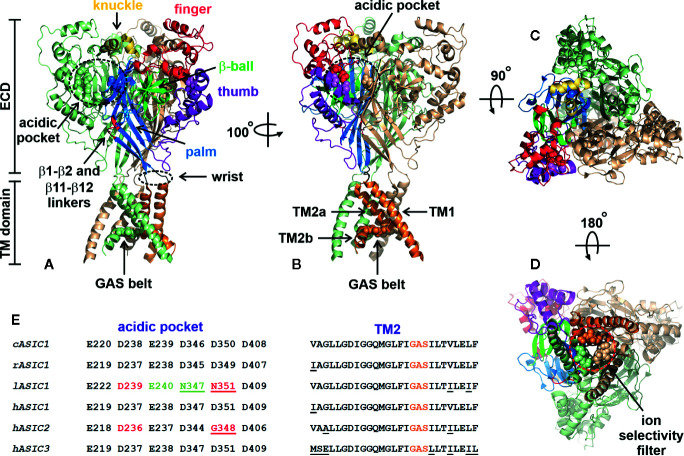
Architecture of chicken ASIC1a channel (PDB code 4NYK). **(A, B)** Side-views of the channel. Two channel subunits are shown by wheat and pale green colors, and the third subunit is colored according its domain structure: the knuckle, finger, b-ball, thumb, palm, and TM part are colored in yellow, dark red, light green, purple, blue, and orange, respectively. Asp and Glu residues forming the acidic pockets (surrounded by black dashed circles) and GAS belts are shown by spheres. The locations of the β1-β2 and β11-β12 linkers are colored by red. **(C)** Top view of the channel. **(D)** View of the channel from an intracellular side. The ion selectivity filter formed by three GAS belts is shown by a red dashed circle. **(E)** Comparison of the residues forming the acidic pocket and TM2 in ASICs of different origin (cASIC1, chicken ASIC1; rASIC1, rat ASIC1; lASIC, lamprey ASIC1; hASIC1,2,3, human ASIC1,2,3).

The structure of an ASIC was first determined in 2007. It was an X-ray crystal structure with 1.9 Å resolution of truncated chicken ASIC1a (cASIC1a), with shortened *N*- and *C*-termini in a low-pH desensitized state (Jasti et al., 2007). Later, in 2009, the structure of desensitized cASIC1a was published with the retained *N*-terminus but still truncated disordered *C*-terminal tail (Gonzales et al., 2009). The cASIC1a structure resembles a bowl formed by three identical subunits (**Figures 2A, B**). Trimers are stabilized by contacts between extracellular domains (ECDs) and transmembrane (TM) helices of adjacent subunits. There is a clear boundary between the ECD and TM part within one subunit formed by a short (3–4 a.a.) linker—also called a “wrist” region—which serves for the signal transduction from ECDs to the TM domain. The ECD of each subunit protrudes above the membrane ~80 Å and in turn has the domain architecture containing the finger, thumb, palm, β-ball, and knuckle domains (**Figure 2A**). Moreover, the proton-, calcium-, chloride-, and other ligand-binding sites are located within ECDs.

Proton-binding sites are located in the middle of ECDs (~45 Å over the membrane) and consist of four spatially close pairs of side-chains of: Asp238-Asp350 and Glu239-Asp346 from the finger and thumb domains of the same subunit, Glu220-Asp408 from the palm domain of the adjacent subunit (these three pairs form the acidic pocket, **Figure 2B**) and Glu80-Glu417 from the palm domain (Jasti et al., 2007). The residues involved in the acidic pocket are highly conservative in ASICs (**Figure 2E**), demonstrating pKa values significantly different from the isolated pKa of aspartate and glutamate residues. Calcium could stabilize the closed, resting state of ASICs at high pH *via* interaction with these pairs, thus recovering the desensitization state (Todorović et al., 2005). On the other hand, these acidic residues have been identified in proton-insensitive ASICs too (Coric et al., 2005), pointing to the possible existence of other proton-binding sites. It was shown that the acidic pocket plays a modulatory function and is subjected to conformational rearrangement upon the activation of a channel, while the pair of Glu80-Glu417 side chains in the palm domain is responsible for acceleration of desensitization and the appearance of sustained current (Vullo et al., 2017). The acidic pocket has extended conformation at high-pH resting and low-pH desensitized states and collapsed conformation at low-pH open state. Collapsed conformation is characterized by approximation of aspartate and glutamate side-chains for the proton-binding, which in turn results in the rearrangement of ECDs and the TM domain to open the channel (Gonzales et al., 2009; Baconguis and Gouaux, 2012; Yoder et al., 2018).

There is a tunnel piercing through the ASIC from the extracellular top to the cytoplasmic bottom (Hanukoglu, 2017). The main function of this vestibule is ion flow from the extracellular environment into the cell. The vestibule is subdivided into upper, central, and extracellular parts. The hydrophobic residues Leu78 and Ile419 (cASIC1) separate the central and extracellular vestibules, forming a trap in a desensitized-like state (Dawson et al., 2012). The extracellular vestibule, playing the role of a cation reservoir, is significantly expanded in the open state compared to closed or desensitized states (Gonzales et al., 2009; Baconguis and Gouaux, 2012; Baconguis et al., 2014; Yoder et al., 2018).

The extracellular part of the vestibule is bounded with the TM domain located in the phospholipid bilayer (**Figure 2**). The TM domain consists of six α-helices (two from each subunit), has an hourglass shape, and plays a dual role, (i) for stabilization and trimerization of the subunits within the channel trimers and (ii) for pore formation and transfer of ions through the cell membrane. The TM part of each subunit is formed by two α-helices: TM1 and TM2. TM1 contacts TM2 of the same subunit, TM1 and TM2 from the adjacent subunits, and the lipid environment, while TM2 lines the channel pore (Gonzales et al., 2009). TM2 consists of two parts (TM2a and TM2b) separated by three residues—Gly443-Ala444-Ser445 (cASIC1)—that are referred to as a GAS belt (**Figures 2A, B**). In the closed gate, TM2 adopts a kinked conformation, forming a pore “gag” with other TM2s from the adjacent subunits. Straightening of the TM2s transfers the pore to the open state with formation of the ion selectivity filter, formed by three GAS belts from the adjacent subunits (Li et al., 2011). The ion selectivity filter is the narrowest part of the pore and serves for the selection of ion types flowing through the channel. The size of the filter (radius ~3.6 Å) correlates well with the radius of hydrated Na^+^ (**Figure 2D**). The TM2 sequence is highly conservative in ASICs, pointing to the similar structure of the pore domain within the whole family (**Figure 2E**).

Presently, the structures of the cASIC1a channel in high-pH resting, low-pH open, and low-pH desensitized states are known (Jasti et al., 2007; Gonzales et al., 2009; Baconguis and Gouaux, 2012; Dawson et al., 2012; Baconguis et al., 2014; Yoder et al., 2018). Two years ago, the structure of the full-length cASIC1a channel was determined by cryo-EM revealing the structural similarity of the full-sized and truncated channels. Based on the cASIC1a structures in all three channel states, the Eric Gouaux group proposed the gating mechanism (Yoder et al., 2018). According to this mechanism, at neutral pH, the channel exists in the closed or resting state. In this state, the acidic pocket is expanded, and the TM domain does not pass the ion flow. When the pH of the extracellular media goes to low values, the acidic pocket changes its conformation from an expanded to a collapsed state, coming closer to carboxyl-carboxylate pairs from the finger and thumb domains and thus binding the protons. This in turn initiates a number of conformational changes in ECDs, with counterclockwise rotation of each subunit, movement of the β1 and β12 strands towards the membrane, and displacement of the TM1 and TM2 helices away from the threefold symmetric axis of the channel. This results in the pore opening and ion flow through the channel. In hundreds of milliseconds (Zhang and Canessa, 2002), the channel switches from its low-pH open state to its low-pH desensitized state, accompanied by reorientation of the β1-β2 and β11-β12 linkers to their initial conformation, and consequently switches movement of TM1 and TM2 back to the center of the pore and closes the channel gate. In other words, the desensitized state of the channel is characterized by simultaneous resting-like conformation of the TM domain and activated-like conformation of the upper half of an ECD with a collapsed acidic pocket and bound protons. This chimeric conformation is reached by rearrangement of the β1-β2 and β11-β12 linkers. Most conformational changes were observed in the β11-β12 linkers, resulting in 9 Å reorientation of the Leu414 residue (cASIC1a) towards the central vestibule. Returning to physiologically high pH values leads to a release of protons from the acidic pocket and its expansion. Thus, the β11-β12 linkers play an important role in channel gating, serving as a bridge between ECDs and the TM domain, within which conformational changes lead to opening or closing of the channel.

In spite of the high relevance of cASIC1a structures in different states and in complexes with various ligands for understanding of the mechanism of channel gating, there are still many blind spots regarding other members of this family. The rat ASIC subunits share ~45–80% of their sequence identities, pointing to a possible difference in the regions responsible for the channel gating. In line with this, the unique Ca^2+^-binding site was recently identified in ASIC3, located in the channel pore (Zuo et al., 2018). Another example of significant structural and functional differences within the ASIC family is lamprey ASIC1, which does not respond to protons. Substitution of only two residues located in the β1-strand and the β1-β2 linker with the corresponding residues Leu77 and Leu85 from rat ASIC1 recovered the proton activation response, suggesting the importance of other structural elements besides the acidic pockets, which significantly differ between rat and lamprey ASIC1s (Li et al., 2010) (**Figure 2E**). Indeed, two residues in the acidic pocket of lamprey ASIC1 that have positions identical to rat Asp345 and Asp349 from cASIC1a are Asn347 and Asn351, respectively. This means that the acidic pockets of lamprey ASIC1 have lower capability to bind protons at pH values close to 5.0. ASIC2a is a pH-sensitive channel, while its splicing variant ASIC2b is not (Schuhmacher et al., 2015). Both variants have identical acidic pockets, albeit with mutated residue corresponding to Asp350 from cASIC1a (**Figure 2E**). This points to the presence of other important structural domains responsible for channel gating. Such domains located in ECDs immediately after TM1 were recently determined for ASIC2a (Schuhmacher et al., 2015).

There are numerous reports about involvement of membrane lipids into control of the spatial structure and function of different receptors and ion channels including GPCRs (Fantini and Barrantes, 2018), the nicotinic acetylcholine receptors (Baenziger et al., 2000), K^+^ and Na^+^ voltage-gated ion channels (Agwa et al., 2018; Jiang, 2019), TRPV1 channels (Morales-Lázaro and Rosenbaum, 2019), TREK channels (Hernández-Araiza et al., 2018), and ENAC channels (Kleyman and Eaton, 2020). Interaction with PIP2 is necessary to open ENaCs (Kleyman and Eaton, 2020), which belong to the same degenerin/epithelial Na^+^ channel (DEG/ENaC) family as ASICs. In line with it, arachidonic acid was shown to potentiate ASIC1a and ASIC3 by direct interaction with the channels in the rat sensory neurons (Smith et al., 2007), and arachidonic acid and lysophosphatidylcholine (16:0) activate ASIC3 at neutral pH and induce pain behavior in rats pointing on lipid-mediated signaling (Marra et al., 2016). At the same time, no data are available about the role of lipids in modulation of ASIC/toxin or ASIC/drug interactions. ASICs also were reported to be involved in mechanosensation (Page, 2005; Lin et al., 2016) demonstrating dual protein functions: sensing both tissue acidosis and mechanical force, although the mechanical gating mechanism of ASICs is still unclear (Cheng et al., 2018). Thus, the membrane environment could be an important modulator of the ASICs activity and the reason for variety of functional properties of the channel subtypes expressed in different cells. Further study of the lipid bilayer role in the ASICs gating is needed.

## Natural Ligands of Acid-Sensing Ion Channels

### Plant Compounds

**Figure 3:**
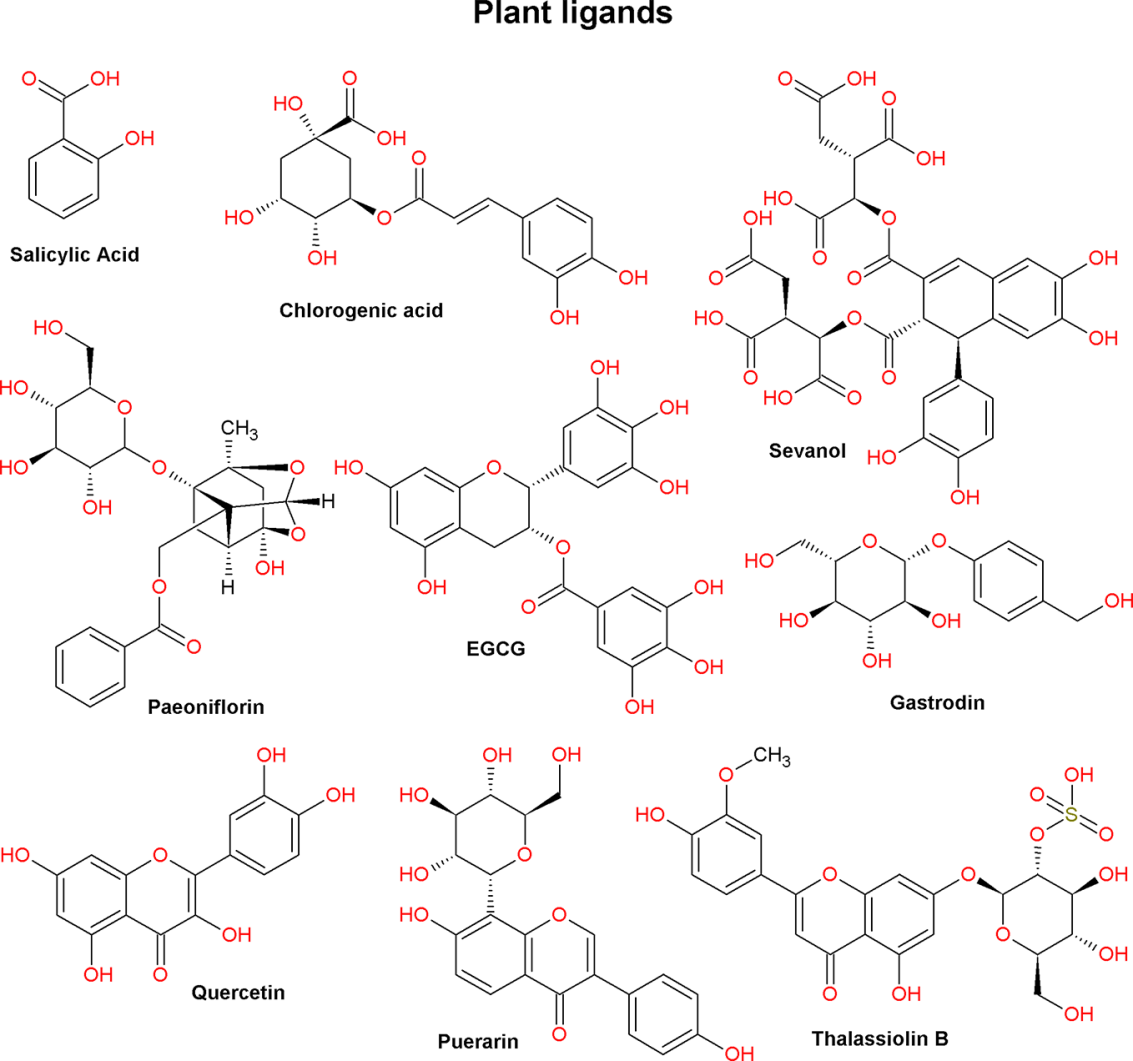
Plant ligands of acid-sensing ion channels (compounds from various chemical classes; see text).

**Figure 4:**
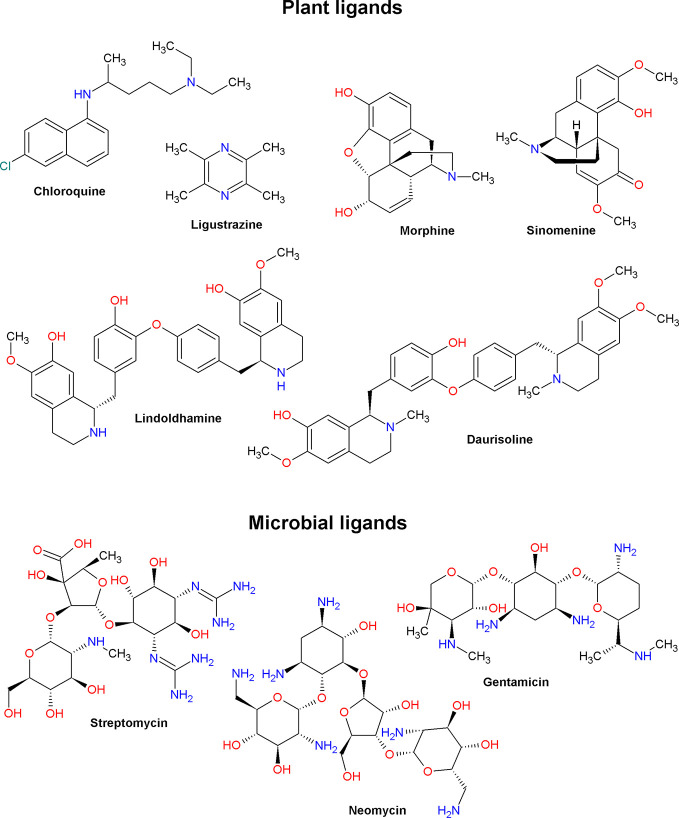
Alkaloid ligands (plant and microbial origin) of acid-sensing ion channels.

Low molecular weight ASIC ligands are represented by molecules belonging to various classes of chemical compounds, ranging from relatively simple pyrazines and polyphenolic acids to more complex glycosides and quinoline alkaloids (**Figures 3** and **4**). Most of these ligands are derived from herbs that are well known for their medical properties and used in the traditional medicine of various nations. The most of described below molecules have several other cellular targets, and their therapeutic effects can be associated not only with the modulation of ASICs. Thus, it is important to keep in view these properties to explain possible auxiliary effects of the compounds, and the biological effect exhibited by these molecules *in vivo* should be considered the result of a complex effect on several targets.

#### 5-Caffeoylquinic Acid

5-caffeoylquinic acid (5-CQA) belongs to a group under the general name chlorogenic acids. It is a phenolic compound, an ester of caffeic acid, and one of the stereoisomers of quinic acid. 5-CQA is abundantly present in various plants included in the human diet (for example, green coffee beans) and is well known for its antioxidant, anti-inflammatory, neuroprotective, and analgesic properties (Zhang et al., 2003; dos Santos et al., 2006; Lapchak, 2007; Li et al., 2008). It was demonstrated on rat dorsal root ganglion (DRG) neurons that 5-CQA is concentration-dependent and reversibly inhibits a pH 5.5-induced current with a half-maximal inhibitory concentration (IC_50_) of 0.235 μM (**Table 1**). To exclude the neuronal acid sensing *via* TRPV1 channels, authors carried out the experiment in the presence of capsazepine (10 μM). Maximal observed inhibitory effect reached 55% that indicated selectivity of 5-CQA to certain ASIC isoforms. The compound also reduces the sensitivity of neurons to various activating pHs, without changing the pH_50_ and Hill coefficient (n_H_) parameters of the pH-dependence activation curve (for a graphic example, see **Figure 1D**, orange dashed curve). In this regard authors conclude that the mechanism of the inhibitory effect of 5-CQA is not associated with a decrease in the affinity of ASIC channels to protons. Local administration of 5-CQA at doses of 1 µM and 10 µM attenuates pain induced by intraplantar injection of 20 μl acetic acid (Qu et al., 2014).

**Table 1:** Effect of plant and microbial ligands *in vitro* on ASICs.

Compound	Trend	Value and object of action	References
(-)-Epigallocatechin gallate	↓	IC_50_ 13.2 μM for mASIC3 in CHO cells	Yan et al., 2019
5-caffeoylquinic acid	↓	IC_50_ 0.235 μM for rASIC in DRG neurons	Qu et al., 2014
Chloroquine	↓	IC_50_ 615.9 μM for rASIC1a in CHO cells	Li et al., 2014
	↑	EC_50_ 425.2 μM for sustained current rASIC3in CHO cells	Lei et al., 2016
Daurisoline	∩	EC_50_ 20 μM for transient current rASIC1a in oocytes	Osmakov et al., 2019b
	↔	EC_50_ 140 μM for rASIC1a in oocytes	
Gastrodin	↓	IC_50_ 0.2 μM for rASIC in DRG neurons	Qiu et al., 2014
Ligustrazine	↓	IC_50_ 270 μM for rASIC in DRG neurons	Zhang et al., 2015
	↓	IC_50_ 97 μM for rASIC1a, 62 μM rASIC1b, 129.4 μM rASIC2a, 239.5 μM rASIC3 in CHO cells	Zhang et al., 2015
Lindoldhamine	↓	IC_50_ 9 μM for rASIC1a oocytes (at pH 6.85 stimulus)	Osmakov et al., 2019c
	Δ	EC_50_ 1.53 mM for hASIC3, 3.2 mM rASIC3 in oocytes	Osmakov et al., 2018
	↑	EC_50_ of 3.8 μM for transient current hASIC3 in oocytes	
Morphine	↓	IC_50_ 2.3 μM for rASIC in DRG neurons	Cai et al., 2014
Neomycin	↓	IC_50_ 45 μM for rASIC in DRG neurons	Garza et al., 2010
Paeoniflorin	↓	IC_50_ 5 μM for rASIC in pheochromocytoma cells	Sun et al., 2011
Puerarin	↓	IC_50_ 38.4 μM for rASIC in hippocampal cells, 9.31 μM for rASIC1a in CHO cells	Gu et al., 2010
Quercetin	↓	IC_50_ 2.4 μM for rASIC1a, 1.3 μM for rASIC2a, 1.8 μM for rASIC3 in CHO cells	Mukhopadhyay et al, 2017
Salicylic acid	↓	IC_50_ 260 μM for rASIC3 in COS cells	Voilley et al., 2001
Sevanol	↓	IC_50_ 353 μM for transient hASIC3, 234 μM for sustained hASIC3 currents in oocytes	Dubinnyi et al., 2012
Sinomenine	↓	IC_50_ 0.3 μM for rASIC1a in CHO cells	Wu et al., 2011
Streptomycin	↓	IC_50_ 30 μM for rASIC in DRG neurons	Garza et al., 2010
Thalassiolin B	↓	IC_50_ 27 μM for rASIC in DRG neurons	Garateix et al., 2011

↓, Inhibition of current; ↑, Potentiating of current; Δ, Activation of current without acidic drop; ∩, Generation of 2nd current component; ↔, Inhibition of steady-state desensitization; IC_50_, half-maximal inhibitory concentration; EC_50_, half-maximal effective concentration; hASIC, human ASIC; rASIC, rat ASIC; mASIC, mouse ASIC.

#### Chloroquine

Chloroquine is an antimalarial drug from the group of 4-aminoquinoline derivatives that is first isolated from an extract of cinchona bark. It has an antiproliferative effect on T cells; reduces the production of several pro-inflammatory cytokines and the innate immune system activation; possesses antiviral, antibacterial, and antifungal effects, and have been successfully used to treat several rheumatological, immunological, and infectious diseases (Plantone and Koudriavtseva, 2018). In the heart, chloroquine block of the inward rectifier K^+^ currents by inhibiting of Kir_2.1_ channel with IC_50_ 8.7 ± 0.9 μM (Rodriguez-Menchaca et al., 2008). One of its known side effects is retinal toxicity (Hobbs et al., 1959). On retinal ganglion neurons and on CHO cells expressing ASIC1a, it was shown that chloroquine dose dependently and reversibly inhibits the amplitude of a pH 6.5-induced current (IC_50_ 615.9 μM) as well as causes a significant delay in peak maximum and desensitization time constant (**Table 1**). This effect was enhanced with an increase in the concentration of Ca^2+^ ions in the extracellular medium and weakened with an increase in the activating acid stimulus. In the presence of chloroquine, the amplitudes of the main parameters of the electroretinogram, such as the b-wave of scotopic 0.01 and photopic 3.0 and vibrational potentials, decreased (Li et al., 2014). In CHO cells expressing ASIC3 channels and in DRG neurons, it was shown that chloroquine dose dependently potentiates the sustained component (EC_50_ of 425.2 μM and n_H_ 3.676) without affecting the transient component of the proton-activated current. This effect also depends on the concentration of calcium in the extracellular medium and weakens with an increase in the activating stimulus. Using site-directed mutagenesis, it was possible to demonstrate that chloroquine can activate ASIC3 channels by binding to a non-proton ligand sensor in the palm domain (Lei et al., 2016). In an *in vivo* “cheek” assay model in mice (Shimada and LaMotte, 2008), chloroquine caused combing, and this effect was attenuated in the presence of an ASIC3 inhibitor (Lei et al., 2016).

#### Lindoldhamine and Daurisoline

Lindoldhamine (LIN) and daurisoline (DAU) are members of the bisbenzylisoquinoline alkaloids group. This is a broad group of biologically active compounds known for their anticancer, antiviral, anti-inflammatory, and neuroprotective properties (Tian and Zheng, 2017). LIN was isolated from an acetic acid extract of the plant *Laurus nobilis* L. On *X. laevis* oocytes expressing human and rat ASIC3 channels, it was shown that LIN is capable of inducing sustained incoming currents with EC_50_ of 1.53 mM and n_H_ 0.93 (for human ASIC3) as well as 3.2 mM and 0.82 (for rat ASIC3) (**Table 1**). Moreover, LIN exerts a potentiating effect on proton-induced currents of human ASIC3, increasing the transient component by more than two times (EC_50_ of 3.8 μM and n_H_ 1.1) as well as inhibiting SSD and restoring the transient component (EC_50_ of 16 μM and n_H_ 1) (Osmakov et al., 2018). LIN also exerts a pH-dependent inhibitory effect on rat ASIC1a channels if a weak stimulus (pH 6.85) is applied. Its strongest inhibitory effect (IC_50_ 9 μM and n_H_ 1.2) weakens with an increase in the acid stimulus. As a result, in the CFA-induced inflammation test, LIN showed a significant anti-inflammatory effect; however, in the acetic acid-induced writhing test, LIN did not show any analgesic effect (Osmakov et al., 2019c).

DAU has a similar structure with LIN but has three more methyl groups (**Figure 4**). DAU is a common compound synthesized by various plants of traditional Chinese medicine. DAU has been shown to have a muscle relaxant and antiarrhythmic effect by inhibiting currents of L-type calcium (active concentration >15 μM) and hERG channels (active concentration >10 μM), respectively (Liu et al., 2010; Liu et al., 2012). On *X. laevis* oocytes expressing the rat ASIC1a channel DAU shows a potentiating effect on a pH 5.5-induced currents, causing the appearance of the second transient component (EC_50_ ~20 μM and n_H_ 1.8) (**Table 1**). DAU also inhibits SSD with an EC_50_ of ~140 μM and n_H_ of 0.8. As a result, DAU causes an acidic shift for both the pH-dependence activation curve and the SSD curve (see **Figures 1C, D**). In general, the mechanism of DAU action can be assumed as competition with protons for desensitization sites on the channel (Osmakov et al., 2019b).

#### (-)-Epigallocatechin Gallate

(-)-Epigallocatechin gallate (EGCG) is an ester of epigallocatechin and gallic acid contained in large quantities in green tea extract. EGCG exhibits pronounced antioxidant activity (Henning et al., 2005), capable to induce apoptosis and inhibit the growth of various types of cancer (Yang et al., 2009). EGCG blocks voltage-gated sodium channel currents at rat hippocampal CA1 neurons (active concentration >100 μM) (Deng et al., 2008) and inhibits the cardiac sodium channel Nav_1.5_ with IC_50_ ~2,1 μM (Amarouch et al., 2020). On CHO cells expressing various isoforms of ASICs, it was shown that EGCG dose dependently and reversibly inhibits the amplitude of the pH 5.0-induced current of mouse ASIC3 with an IC_50_ of 13 μM (Yan et al., 2019). It should be noted that the specificity of the action was checked on isoforms belonging to different species, namely human ASIC1a, rat ASIC1b and 2a, and mouse ASIC3. Thus, this leaves open the question of the species specificity of the action of EGCG. A study of the structure–activity relationship showed that the presence of the gallate part, the presence of the 3-hydroxyl group on the pyrogallol part, and the chirality of the pyrogallol part play an important role in the activity of the molecule. In a hind paw licking test in mice, it was demonstrated that prior local administration of EGCG (100 µM) attenuates (0.6%) acetic acid induced pain-related behaviors (Yan et al., 2019).

#### Gastrodin

Gastrodin, a gastrodigenin glycoside, is the main bioactive component of the *Gastrodia elata Blume* orchid extract used in traditional Chinese medicine. Gastrodin is known to inhibit of the M-type K^+^ currents in neurons with IC_50_ 19.4 µM (Yang et al., 2019). It has anticonvulsant and analgesic properties alleviating migraine and trigeminal neuralgia, as well demonstrates a neuroprotective effect in ischemia (Kumar et al., 2013; Zeng et al., 2006). On rat DRG neurons, it was demonstrated that gastrodin reversibly and concentration dependently reduced the amplitude of pH 5.5-induced current with an IC_50_ of ~0.2 μM (**Table 1**). At the same time, like 5-CQA (see above), gastrodin in the presence of capsazepine (10 μM) reduces the sensitivity of neurons to various activating pHs but did not change the main parameters (pH_50_ and n_H_) of the pH-dependence activation curve. In acid- and formalin-induced pain-related behaviors in rats, gastrodin, having previously been locally administered into the paw (up to concentration of 10 μM), shows analgesic and anti-inflammatory effects (Qiu et al., 2014).

#### Ligustrazine (Tetramethylpyrazine)

Ligustrazine, a member of the alkylpyrazine group, is a bioactive component found in the extract of the plant *Ligusticum chuanxiong* Hort., used in traditional Chinese medicine. Its neuroprotective, vasodilating, and cardioprotective effect has been described (Liu et al., 1990; Zhou et al., 2004; Cheng et al., 2007). Its inhibitory activity on L-type calcium current of myocytes with IC_50_ varied in 88.19–200 μM range depending to experimental conditions was found as well (Zou et al., 2001; Ren et al., 2012). Also in micromolar range ligustrazine inhibits the transient component of pH 5.0-induced current with an IC_50_ of ~270 μM (**Table 1**) in rat DRG neurons as well as decreases the number of action potentials evoked by acidosis. The specificity of ligustrazine pH-dependent inhibition was studied on CHO cells expressing various asic isoforms, and the following results were obtained: IC_50_ of 97 μM for ASIC1a, 62 μM for ASIC1b, 129.4 μM for ASIC2a, and 239.5 μM for the ASIC3 current. In this instance, ligustrazine does not change the channels’ affinity to protons, but reduces their opening efficiency. Ligustrazine repressed the ST segment (at doses of 3 and 10 mg/kg) and coronary artery occlusion-related T-wave (at doses of 20 and 30 mg/kg) in rat angina models and inhibited the myocardial infarction at doses of 3 mg/kg, thus reducing the necrotic area. Ligustrazine also showed a significant analgesic effect in the acetic acid-evoked pain response in rats (Zhang et al., 2015).

#### Paeoniflorin

Paeoniflorin is a monoterpene glycoside. It is one of the main bioactive components in the root extract of the peony *Paeonia lactiflora*. Antidepressant-like, immunostimulating, anticancer, and pro-apoptotic effects have been shown for this compound (Chen et al., 2012; Hu et al., 2013; Qiu et al., 2013). Paeoniflorin produces an inhibition of L-type calcium current in NG108-15 cells with IC_50_ 14 μM (Tsai et al., 2005) and inhibits Cav_1.2_ channels (the active concentration >50 μM) (Song et al., 2017). On rat pheochromocytoma cells, paeoniflorin demonstrates a cytoprotective effect. This effect is associated with ASICs’ inhibition since paeoniflorin dose dependently blocks pH 6.0-induced currents (IC_50_ ~5 μM) and inhibited these channels’ expression in the cells, which was estimated by both RT-PCR and ASIC-specific antibody labelling. As a result, it was found that the activity of paeoniflorin leads to increased autophagic degradation of α-synuclein and can serve as evidence of the participation of ASICs in the development of Parkinson’s disease (Sun et al., 2011).

#### Quercetin

Quercetin is a pentahydroxyflavone widely distributed in many vegetables and fruits such as tomato, onion, citrus fruit, and a number of berries. It has anti-inflammatory, anticancer, cardio and neuroprotective, and antibacterial and antiviral properties (Anand David et al., 2016). It can inhibit heart Na_V1.5_ channels with IC_50_ of 19.4 μM (Wallace et al., 2006) and activate vascular smooth muscle L-type calcium channels with EC_50_ ~5 μM (Saponara et al., 2002). On CHO cells expressing various isoforms of rat ASICs, quercetin was shown to equally inhibit pH-induced currents with an IC_50_ of 2.4, 1.3, and 1.8 μM for ASIC1a, ASIC2a, and ASIC3, respectively (**Table 1**). Quercetin prevents a pH 6.0-induced increase of intracellular Ca^2+^ concentration in HEK-293 cells and significantly reduces their mortality. A molecular docking approach with rat ASIC1a site-directed mutagenesis detected the possible involvement of the channels’ central vestibule residues Arg369 and Glu416 in the interaction with quercetin (Mukhopadhyay et al., 2017).

#### Puerarin

The flavonoid consisting of C-glycosyl and hydroxyisoflavone parts is the main bioactive constituent of the leguminous plant *Pueraria lobata* (Willd.) Ohwi extract. Its pharmacological activity as an anti-inflammatory, analgesic, neuroprotective, anticancer, and antioxidant molecule is known (Zhou et al., 2014). Puerarin inhibits potassium channels Kir_2.1_ (IC_50_ 1.27 mM) and Kv_7.1_ (IC_50_ ~55 μM) (Xu H. et al., 2016), and the resting Na_v_ channels of DRG neurons (IC_50_ 481 μM) (Zhang et al., 2019). Studies on rat hippocampal cells, as well as on CHO cells expressing ASIC1a, showed that puerarin has an inhibitory effect with IC_50_ and nH values of 38.4 μM and 5.97 (hippocampal cells) as well as 9.31 μM and 8.18 (CHO cells) (**Table 1**). Moreover, acceleration of desensitization in the presence of puerarin was observed in both systems because cytoprotector puerarin (100 µM) significantly reduces the mortality of hippocampal neurons exposed to the acidic (pH 6.0) solution (Gu et al., 2010).

#### Thalassiolin B

Another flavonoid consisting of chrysoeriol and O-glycosyl sulfate moieties abundantly presented in the sea grass *Thalassia testudinum* extract. Thalassiolin B was initially known as the antioxidant (Regalado et al., 2009). Later, it was shown that thalassiolin B inhibits the transient component of the proton-induced current in rat DRG neurons with an IC_50_ of 27 μM (**Table 1**). In a formalin test in mice, thalassiolin B (100 mmol/kg) alleviated pain behavior, reducing the number of licks during the first- and second-phase nociception (Garateix et al., 2011).

#### Salicylic Acid

Salicylic acid, or 2-hydroxybenzoic acid, is an important signaling component in plant immunity (Marek et al., 2010). This compound is well known for its anti-inflammatory, topical antibacterial, and cosmetic properties (Arif, 2015). It was shown that salicylic acid is able to inhibit the sustained component of current through ASIC3 channels expressed in COS cells with an IC_50_ of 260 μM, as well as the ASIC3/ASIC2b heteromeric current at a concentration of 500 μM (**Table 1**) (Voilley et al., 2001).

#### Sevanol

Sevanol (or 9,10-diisocytril ester of epiphylic acid) belongs to the group of polyphenolic compounds called lignans. Sevanol was isolated from an acetic acid extract of *Thymus armeniacus*, whereas it was absent in extracts of other representatives of this genus (Osmakov et al., 2015). On *X. laevis* oocytes expressing the human ASIC3 channel, it was shown that sevanol is able to inhibit both components of the ASIC3 current. The transient component of the current is completely inhibited (IC_50_ of 353 μM), whereas the sustained component is inhibited by only 45% (IC_50_ of 234 μM) (**Table 1**). Sevanol also inhibits the rat ASIC1a channel but with less efficacy (Dubinnyi et al., 2012). In models of acetic acid-induced writhing and CFA-induced thermal hyperalgesia tests, sevanol showed dose-dependent (range from 0.001 to 10 mg/kg), pronounced analgesic and anti-inflammatory effects (Andreev et al., 2018).

#### Morphine

Morphine—a morphinane alkaloid and a tertiary amino heteropentacyclic compound—is the most abundant opiate of the opium poppy (Papaver plant). It is well known as an analgesic, anxiolytic, and vasodilator drug with a number of serious side effects (including addiction) that are a result of its action on opiate receptors (Pathan and Williams, 2012). Studies on rat DRG neurons in the presence of capsazepine showed that morphine reversibly and dose dependently inhibits the pH-induced currents of ASICs with an IC_50_ of 2.3 μM (**Table 1**). The pH dependence of activation does not change significantly in the presence of morphine. Further pharmacological analysis showed that the effect of morphine on neurons is mediated by µ-opioid receptors and depends on the cAMP signaling pathway. In an acid-induced pain test on rats, preliminary local administration of 1–10 μM morphine causes a significant decrease in the number of flinches of the hind paw, but this analgesic effect disappears in the presence of an opioid receptor-inhibitor naloxone (Cai et al., 2014). Morphine can also be synthesized in mammals (Poeaknapo et al., 2004), and it was shown that intermediates in this synthesis pathway—isoquinoline alkaloids tetrahydropapaveroline and reticuline—can directly potentiate ASIC3 channels (Osmakov et al., 2017).

#### Sinomenine

Sinomenine, by the chemical structure related to the family of morphinane alkaloids, is one of the main biologically active components of the extract of the medicinal plant *Sinomenium acutum*. It has been shown that sinomenine possessed an anti-inflammatory function and regulated the secretion of multiple inflammatory cytokines and monocyte/macrophage subsets (Liu et al., 2018), and it has also been used in the clinic for the treatment of rheumatoid arthritis (Xu et al., 2008). On rat cortical neurons, as well as on CHO cells expressing recombinant channels, it was shown that sinomenine directly and dose dependently inhibits ASIC1a channels with an IC_50_ of ~0.3 μM (**Table 1**). Sinomenine (1 μM) significantly reduces 30 mM KCl, and acidosis-induced increases in intracellular Ca^2+^ concentration, suggesting the inhibitory effect of sinomenine on L-type calcium channels. In the cerebral ischemic insult model, sinomenine exerted a neuroprotective effect and improved brain functional recovery (Wu et al., 2011).

### Microbial Compounds

#### Aminoglycosides (Streptomycin, Neomycin, and Gentamicin)

Aminoglycosides are a group of broad-spectrum antibiotics that exhibit bactericidal action by inhibiting protein biosynthesis. Their structure is based on amino sugars linked together, as well as with aminocyclitol rings, *via* a glycosidic bond (Krause et al., 2016). They also showed a blocking effect on Na^+^- and Ca^2+^-channels (Zhou and Zhao, 2002), as well as on transient receptor potential V1 (TRPV1) channels (Raisinghani and Premkumar, 2005). In DRG neurons, streptomycin (ST) and neomycin (NEO) reversibly but not completely decrease the amplitude of pH 6.1-induced currents with IC_50_ ~30 μM and n_H_ 1.3 for ST and IC_50_ ~45 µM and n_H_ 1.7 for NEO (**Table 1**. In this case, ST and NEO, as well as gentamicin, have a slowing effect on the desensitization process, and this effect is enhanced with a decrease in the concentration of Ca^2+^ ions in the extracellular medium. On HEK-293 cells expressing human ASIC1a, only ST shows an incomplete inhibitory effect, without acting, however, on the kinetics of desensitization (Garza et al., 2010).

### Animal Venom Toxins

To date, ASIC peptides’ modulators have been extracted from the venoms of spiders, snakes, sea anemones, and wasps. Taken together they differ in structure, subtype specificity, and the mode of action onto the channels. Although many of the toxins are not highly specific to ASICs over other cellular targets, a lot of them have adequate affinity for these ion channels. Even if the measured affinity to the channel/receptor is in the micromolar range, this does not exclude such molecules from the list of promising drug seeds (Kozlov, 2018).

#### RF-Amide Peptides

**Figure 5:**
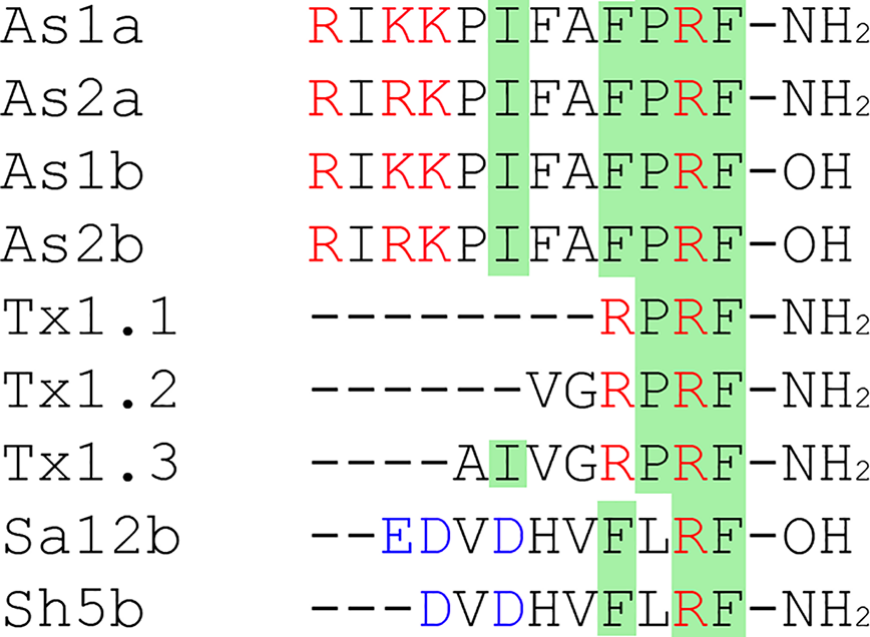
RF-amide peptides family. The same residues are highlighted in green, negatively charged residues Asp and Glu are written in blue, and positively charged residues Lys and Arg are written in red.

Cono-RFamides (CNFs) are a group of amides, isolated from cone snails’ venom, with particular characteristics: the short length, the *C*-terminal Arg–Phe–NH_2_ (RFa) motif, and the lack of cysteine residues (linear peptide) (**Figure 5**). Natural analogues with non-amidated *C*-terminus detected in venoms were inferior in their ability to potentiate ASIC channels.

Two conorfamides As1a and As2a and their non-amidated forms As1b and As2b from *Conus austini* differ by the mutation in the third residue (Lys in As1a and As1b, Arg in As2a, As2b). It was shown that these compounds modulate proton-induced rat ASIC1a and ASIC3 currents by slowing channel desensitization following a sustained current inducing but they have no effect on homomeric rat ASIC1b or ASIC2a, expressed in *X. laevis* oocytes (Jin et al., 2019). Amidation of *C*-termini is essential for the peptide activity, since non-amidated As1b and As2b show only weak inhibition of transient currents. The most active As2a potentiates ASIC1a with an EC_50_ of 10.9 μM by the mechanism of the shift of the channel-desensitization constant (gray curve on the **Figure 1A**), and the resulting current has an unusual shape with a large amplitude. Similarly, this peptide affects the ASIC3 subtype. The second interesting peptide As1a, up to 200 µM, has a moderate effect on the sustained current generation for rASIC1a and rASIC3.

Another group of CNFs isolated from the venom of *Conus textile* is represented by three peptides (Tx1.1, Tx1.2, Tx1.3) consisting of 4, 6, and 8 amino acid residues, respectively. These CNFs show effects on ASIC3 currents like As2a peptide but also increase the transient current amplitude. The shortest peptide (CNF-Tx1.1) also potentiates the currents of heterotrimers ASIC1a/3, ASIC1b/3, ASIC2a/3, and ASIC2b/3 and has a higher affinity to homotrimer than CNF-Tx1.2. The potentiating effect of CNF-Tx1.1 is implemented by several mechanisms: shifts proton affinity of ASIC3 to higher pH for potentiation (green curve on the **Figure 1D**); lowers pH for desensitization (green curve on the **Figure 1C**); and increases the transient current amplitude 1.48-fold (blue curve on the **Figure 1D**). Intramuscular injection of this CNF in mice increases acid-induced muscle pain (Reimers et al., 2017).

Linear peptides that activate ASIC are not only found in marine snail venoms. Recent studies have described RF-amide modulators of ASICs in wasp venom. Peptide Sa12b extracted from the venom of solitary wasp *Sphex argentatus argentatus* reversibly and pH-independently inhibits the ASIC currents of rat DRG neurons with an IC_50_ ~81 nM when it is applied before the activation stimulus. Peptides’ co-application with stimulus does not produce any significant alteration in acid-induced currents, which indicates the peptides binding with the channel closed state. In the same work another peptide Sh5b with a very similar structure (**Figure 5**), purified from the venom of wasp *Isodontia harmandi*, changes the ASIC current parameters in DRG neurons insignificantly (Hernández et al., 2019).

#### Polypeptide Toxins

**Figure 6:**
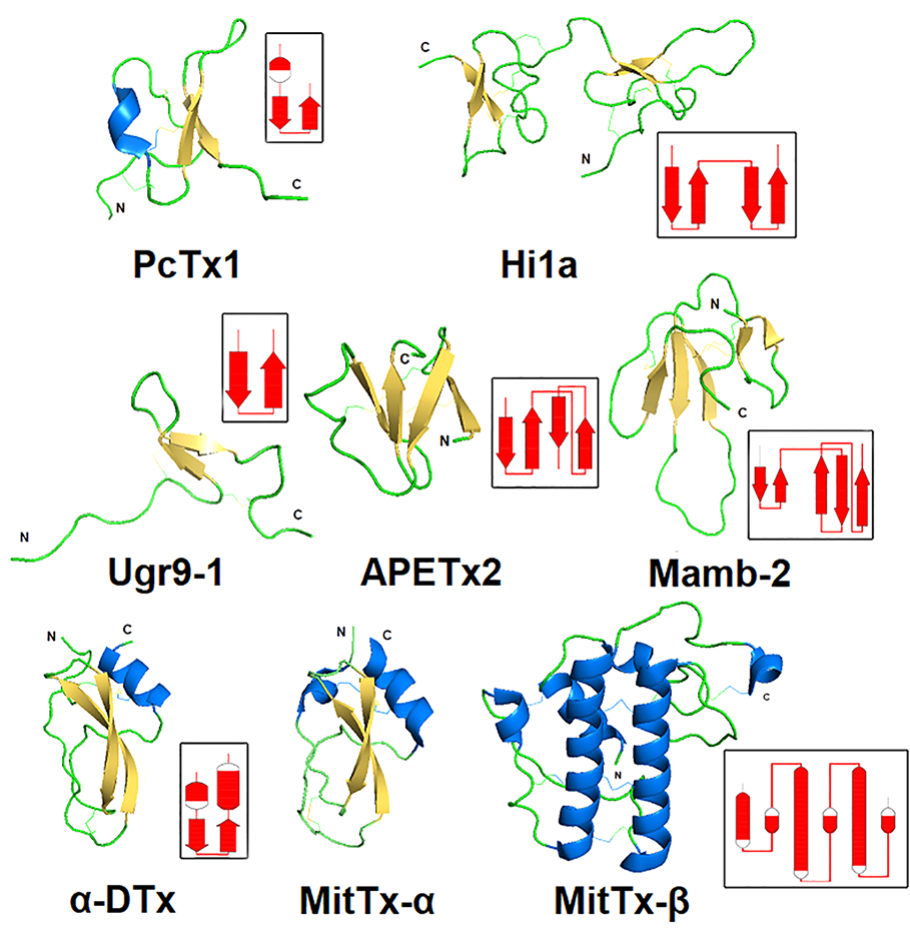
Spatial structure of animal polypeptide toxins. The structures drawn according to the following PDB data: PcTx1 (2KNI), Hi1a (2N8F), Ugr9-1 (2LZO), APETx2 (2MUB), Mamb-2 (2MFA), α-DTx (1DTX), and MitTx-α, MitTx-β (4NTW). The distribution scheme of the secondary structure elements for each toxin is shown next to the 3D structure as an inset.

Polypeptide animal toxins are able to modulate the activity of ASICs with higher affinity at nanomolar concentrations. Historically, two toxins, psalmotoxin (PcTx1) and APETx2, became the basis of numerous scientific works in which they were used as molecular tools to study the function of ion channels ASIC1 and ASIC3 in living organisms. Later, other toxins were discovered, but so far all of them have been isolated from venomous animals such as spiders, sea anemones, and snakes. Toxins differ in their size and spatial organization, which was obtained by the NMR technique as a solution for a number of polypeptide toxins (**Figure 6**), as well as by X-ray structural analysis in the complex with cASIC1a (see section below).

#### PcTx1-Related Toxins

**Figure 7:**
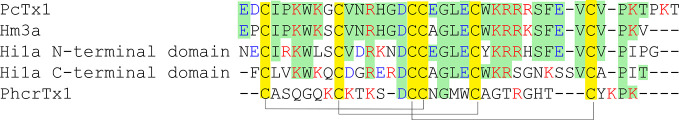
Alignment of primary structures of PcTx1-like toxins. Cysteine residues are highlighted in yellow, and lines represent disulfide bridges’ formation. The sequence residues similar to PcTx1 are highlighted in green, negatively charged residues Asp and Glu are written in blue, and positively charged residues Lys and Arg are in red.

A 40-amino-acid-long peptide Psalmotoxin 1, isolated from the spider *Psalmopoeus cambridgei*, shares the folding named «inhibitor cystine knot» (ICK), which is a structural motif characterized by a triple-stranded anti-parallel β-sheet connected by three disulfide bonds forming a knotted core (**Figure 6**). This ICK motif is a major type of spider toxin organization, so distribution of the Cys residues like those presented in (**Figure 7**) is utilized for toxins’ prediction from modern big data (Kozlov et al., 2005; Kozlov and Grishin, 2011).

PcTx1, a highly basic polypeptide (pI 10.38), was discovered as the first high-affinity and highly selective pharmacological agent to ASIC1a (Escoubas et al., 2000). PcTx1 acts like a selective reversible inhibitor of rat ASIC1a currents (IC_50_ of 0.9 nM), fully blocking it at 10-nM peptide concentration (Escoubas et al., 2000), and of human ASIC1a (IC_50_ of 3.2 nM) (Cristofori-Armstrong et al., 2019). The inhibition mechanism of PcTx1 is increasing its apparent affinity for H^+^ through the desensitization of the channel (red curve on the **Figure 1C**) (Chen et al., 2005). It also inhibits mouse ASIC1a/2b (IC_50_ of 2.64 nM) (Sherwood et al., 2011) and rat ASIC1a/2a (Joeres et al., 2016). At the same time, PcTx1 potentiates the ASIC1b isoform with an EC_50_ of ~100 nM (Chen et al., 2006). This toxin has been used many times to study the properties of channels from wild neurons and heterologously expressed channels. In particular, interesting results were obtained in showing that ASIC1a subtypes play an important role in retinal activity (Ettaiche, 2006). Also, due to the successful selective inhibition of the ASIC1a subtype, it was shown that ASIC1b is involved in the development of muscle pain (Chang et al., 2019) and that the ASIC3 subtype is responsible for postoperative pain (Deval et al., 2011).

One more ASIC-active toxin that has an 82% resemblance to PcTx1 (shortened at three *C*-terminal residues and five residues substitution) was named π-TRTX-Hm3a (Hm3a). It was extracted from the venom of a Togo starburst tarantula (*Heteroscodra maculata*) (Er et al., 2017). In general, Hm3a shares the pharmacological profile of PcTx1. It inhibits acid-evoked currents of rASIC1a expressed in *X. laevis* oocytes, with an IC_50_ of 2.6 nM. It potentiates currents of homomeric rASIC1b and heteromeric rASIC1a/ASIC1b with an EC_50_ of 46.5 and 17.4 nM, respectively. This peptide does not show any effect on homomeric rASIC2 or rASIC3. Hm3a also inhibits human ASIC1a (IC_50_ of 39.7 nM) and potentiates human ASIC1b (EC_50_ of 178.1 nM), being ~30-fold and ~3.8-fold less potent to rat isoforms respectively. It was shown that Hm3a is more stable and resistant to thermal, chemical, and enzymatic degradation than PcTx1, becoming a more attractive tool for studying ASICs *in vivo* (Er et al., 2017).

A disulfide-rich polypeptide Hi1a consisting of 75 residues was found in the venom of the Australian funnel-web spider *Hadronyche infensa*. The structure represents two PcTx1-like ICK domains with a short linker connecting them. The *N*- and *C*-terminal moieties have 62 and 50% similarity to the PcTx-1 sequence, respectively, leading to a suggestion that this peptide originated through duplication of a gene encoding PcTx1-like toxin (Chassagnon et al., 2017). Hi1a equipotent inhibits rat and human ASIC1a expressed in *X. laevis* oocytes (IC_50_ of 0.40 and 0.52 nM, respectively), but unlike PcTx1, it does this incompletely even at saturating peptide concentrations. Inhibition of the rASIC1a subtype is more than 2,000 times more potent over other subtypes, with the toxin having no effect on rASIC2a and rASIC3 up to 1 μM and weakly potentiating rASIC1b. Toxin Hi1a has slow current-inhibition reversibility (τ_off_ = 14.2 min for rASIC1a, 31.8 min for hASIC1a after application 10 nM of Hi1a), with ~40% recovery of the current amplitude after a 30-min washout, which was not reported for any ASIC modulators before. Moreover, Hi1a shows less pH-dependent inhibition, which means a small acidic shift (0.18 pH units at 5 nM for hASIC1a) in contrast to PcTx1. *In vitro* tests on primary oxidatively stressed neuron/astrocyte cultures and *in vivo* tests on a rat-focal cerebral ischemia model showed even greater neuroprotective efficacy of Hi1a over PcTx1 (Chassagnon et al., 2017).

This structural group also includes the ICK cnidarians peptide PhcrTx1 purified from the sea anemone *Phymanthus crucifer*. Like PcTx1, this toxin is a basic peptide (pI = 10.89), but its overall homology with PcTx1 is a negligible (only 28%). The peptide reversibly inhibits the transient component of pH 6.1-induced ASIC currents in rat DRG neurons with IC_50_ ~100 nM without significantly affecting the time course of desensitization and with no effect on the sustained component (Rodríguez et al., 2014).

#### APETx2-Related Toxins

**Figure 8:**
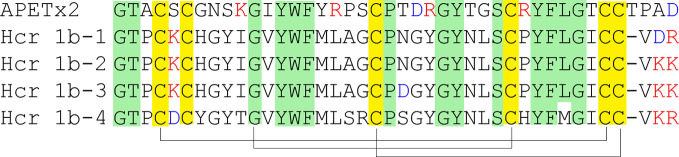
Alignment of primary structures of APETx2-like toxins. Cysteine residues are highlighted in yellow, and lines represent disulfide bridges’ formation. The residues similar to APETx2 structure are highlighted in green, negatively charged residues Asp and Glu are in red, and positively charged residues Lys and Arg are in blue.

Currently, five peptide modulators of ASICs attributed to the structural class 1b of sea anemone toxins (Kozlov and Grishin, 2012) have been described (**Figure 8**). Polypeptide APETx2 isolated from the venoms of the sea anemones *Anthopleura elegantissima* was positioned for a long time as the specific inhibitor of the ASIC3 subtype. The structure of this small 42-amino-acid-long polypeptide has a large number of positively charged amino acid residues like PcTx1 (pI = 9.59). According to the 3D structure resolved by the NMR technique, APETx2 is a β-defensin-like peptide consisting of a compact disulfide-bonded core from a four-stranded beta-sheet, cross-linked by three disulfide bridges (**Figure 6**) (Chagot et al., 2005).

APETx2 rapidly and reversibly blocks homotrimeric ASIC3 and heterotrimeric channels containing ASIC3 without any effect on homomeric ASIC1a. The IC_50_ values are 63 nM for rASIC3 expressed in *X. laevis* oocytes and 175 nM for human ASIC3 expressed in COS cells. Heteromeric ASIC1a/3 and ASIC1b/3 in COS cells are also inhibited by APETx2 but with less affinity (IC_50_ of 2 and 0.9 µM, respectively). The ASIC1a/3 current can only be partly inhibited (~60% by 3 µM concentrations of APETx2). A better result was obtained for ASIC2b/3 heteromers, and the transient current of which was inhibited to approximately 36% of the control amplitude with an IC_50_ of 117 nM (Diochot et al., 2004; Logashina et al., 2020). It was shown that APETx2 potentiates the activity of rASIC1b at concentrations 30- to 100-fold higher than it inhibits rASIC3 homomers, causing an increased current’s desensitization, with no effect on the rise time. Moreover, APETx2 potentiates rASIC2a currents using a different method (decrease in the current rise time, with no effect on desensitization time). In both cases, APETx2 appears to provide stabilization of the open state for rASIC1b and rASIC2a (Lee et al., 2018). Furthermore, the toxin weakly inhibits different potassium channels and TTX-resistant currents of DRG neurons (Diochot et al., 2004; Blanchard et al., 2012) but can reduce the Na_V1.2_ and Na_V1.8_ current in oocytes above 50% in nanomolar concentrations (IC_50_ of 114 nM for Na_V1.2_ and 55 nM for Na_V1.8_) (Peigneur et al., 2012). At micromolar concentrations, APETx2 inhibits hERG (IC_50_ of 1.21 μM) reversible with a maximal inhibition of 54%, and this experimental fact would seriously limit its potential as an analgesic (Jensen et al., 2014).

In the venom of the sea anemone *Heteractis crispa*, several toxins were found with related structures to APETx2 and inhibitory activity to ASICs (**Figure 8**). Between themselves, the similarity of these toxins is high (1–9 substitutions from 41 total residues), but their activity to two main isoforms ASIC1a and ASIC3 is different. The most represented in venom peptide π-AnmTX-Hcr1b-1 reversibly inhibits transient component of human ASIC3 currents expressed in *X. laevis* oocytes with an IC_50_ of 5.5 μM (Kozlov et al., 2012). Otherwise, Hcr1b-2 inhibits currents through rat ASIC1a (IC_50_ of 4.8 μM) more prominently than ASIC3 currents (IC_50_ of 15.9 μM). Such possibilities in the reduction of ASIC3 activity in PNS and ASIC1a activity in CNS make this peptide the prospective candidate for analgesia investigation, and Hcr1b-2 showed an analgesic activity *in vivo*, significantly reducing the number of writhing of experimental animals in acetic acid-induced writhing test (Kalina et al., 2018). The peptide Hcr 1b-3, having one residue substitution to the Hcr1b*-*2 sequence, keeps the same effectiveness for ASIC1a and ASIC3 inhibition with an IC_50_ of 4.95 μM and 17 μM, respectively. The more structurally diverse toxin Hcr1b*-*4 is capable of inhibiting the rASIC1a with an IC_50_ of 1.25 μM, but with the same potency of EC_50_ of 1.53 μM, it potentiates rASIC3 currents (Kalina et al., 2020). The diverse activity of Hcr1b*-*4 for two different ASIC isoforms with apparently the same affinity makes it a very interesting tool for structural and bimolecular research.

#### Toxin Ugr 9-1

Peptide π-AnmTXUgr 9a-1 (Ugr 9-1) isolated from the venom sea anemone *Urticina grebelnyi* consists of 29 amino acid residues and belongs to a structural class 9a (Kozlov and Grishin, 2012). Its spatial structure named “boundless β-hairpin” (BBH) was resolved (**Figure 6**). It is a twisted β-hairpin without interstrand disulfide bonds connected by two S-S bridges, with *C*- and *N*-terminal tails. The peptide shows the reversible inhibition effect on human ASIC3 expressed in *X. laevis* oocytes. It completely blocks the transient component with an IC_50_ of 10 µM, and only by 48% inhibits the sustained component with an IC_50_ of 1.44 µM (Osmakov et al., 2013). Intramuscular or intravenous injection of Ugr 9-1 (0.01–1 mg/kg) produced a significant analgesic effect in the acid-induced pain model and the complete Freund’s adjuvant-induced thermal hyperalgesia test (Osmakov et al., 2013; Andreev et al., 2018).

#### Snake Toxins

**Figure 9:**
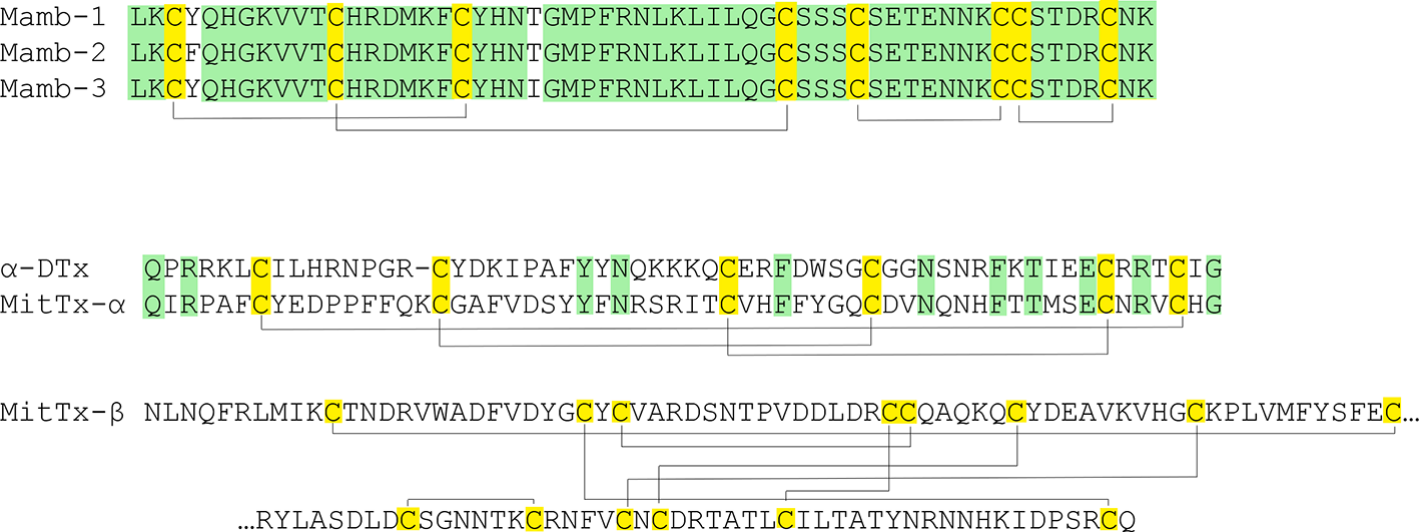
Snake toxins active onto ASICs. Cysteine residues are highlighted in yellow, and lines represent disulfide bridges’ formation. The similar residues are highlighted in green.

Various by structure snake toxins are able to modulate the activity of ASICs. Only a limited number of species produce toxins affecting proton-activated ion channels, and it is obvious that these channels are not primary target for their venom action. The sequences of all currently known toxins are summarized in **Figure 9**, while the spatial organization of toxins is shown in **Figure 6**.

One group of polypeptides was extracted from the venoms of different poisonous snakes. These include mambalgin-1 and mambalgin-2 isolated from the venom of the black mamba *Dendroaspis polylepis*, as well as mambalgin-3 from the venom of the green mamba *Dendroaspis angusticeps*. Toxins utilized a very common fold for snake venom compounds called three-finger toxins, and they contain 57 amino acid residues and 4 disulfide bonds. The sequences of peptides are identical and differ in one residue substitution (mamb-1 to mamb-2/Tyr to Phe in 4th position and mamb-1 to mamb-3/Thr to Ile in 23rd position) (Diochot et al., 2012). According to the authors, despite the similar core, the structure of mamb-2 stands out amongst common short three-finger toxins of a snake as a result of its shortened first and third fingers and elongated middle finger. Mambalgin-1 and mambalgin-2 reversibly inhibit homomeric rASIC1a and heteromeric rASIC1a/2a and rASIC1a/2b expressed in *X. laevis* oocytes with an IC_50_ of 55, 246, and 61 nM, respectively. They act as a gating modifier toxin by decreasing the apparent proton sensitivity of activation (red curve on the **Figure 1D**) and by slightly increasing the apparent proton sensitivity for inactivation (red curve on the **Figure 1C**) (Diochot et al., 2012). Also, they inhibit rASIC1b and rASIC1a/1b with an IC_50_ of 192 and 72 nM, respectively. These peptides inhibit human ASIC1a (IC_50_ of 127–580 nM) (Wen et al., 2015). In addition, it was shown that they inhibit ASIC currents in the spinal cord and sensory and hippocampal neurons but that mambalgins lack any effect on ASIC2a, ASIC3, ASIC1a/3, and ASIC1b/3 (Diochot et al., 2012).

The double-chain toxin MitTx was isolated from the venom of the Texas coral snake (*Micrurus tener tener*). Two non-covalently associated subunits of Kunitz-type protease inhibitors MitTx-α and phospholipase-A2-like MitTx-β are combined in one active molecule to function as a strong and selective agonist for ASICs. The current induced by the toxin is not desensitized but is inhibited by ASICs’ selective inhibitors. The selectivity of MitTx depends on pH, whereas at the neutral pH values, the toxin potentiates the predominant ASIC1 subtype but changes selectivity towards ASIC2a at a pH below 6.5. For the homomeric channels expressed in *X. laevis* oocytes, EC_50_ of 9.4 nM for rat ASIC1a, 23 nM for rat ASIC1b, 36 nM for rat ASIC2a, and 830 nM for rat ASIC3 were calculated. It did not show any effect on 2b or 4 subtypes. Heteromeric rASIC1a/2a and rASIC1a/3 expressed in CHO cells are only mild and weakly activated (Bohlen et al., 2011). Injection into the hind paw of wild-type mice resulted in a painful sensation determined by licking the paw. The time of licking was reduced in ASIC1a-knockout mice, meaning that pain-related behavior was mostly linked to the interaction of the toxin with this channel subtype (Bohlen et al., 2011).

One more toxin with Kunitz-type protease inhibitors fold - α-dendrotoxin (α-DTx) is also ASICs modulating peptide. Similar to mambalgin-3, this toxin was isolated from the Eastern green mamba *Dendroaspis angusticeps*, and its main biological targets are voltage-gated potassium channels (specifically of Kv_1.1_, Kv_1.2_ and Kv_1.6_ with IC_50_ of 9.4, 0.38, and 9 nM, respectively) (Tabakmakher et al., 2019). In contrast to MitTx described above, α-DTx reversibly inhibits the transient component of pH 6.1-induced ASIC currents in rat DRG neurons with an IC_50_ of 0.8 µM without remarkable impact on the current desensitization rate, and at 3 µM concentration, α-DTx also inhibits the sustained component (Báez et al., 2015).

## Structure–Function Relationships in Peptide Toxins and ASICs

**Figure 10:**
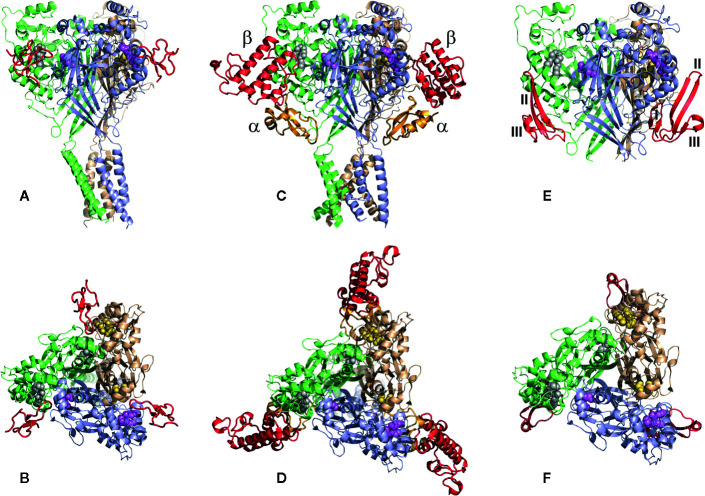
The structures of the cASIC1a complexes with different peptide toxins. The channel subunits are shown by wheat, pale green, and blue colors. Asp and Glu residues forming the acidic pockets in corresponding subunits are shown by spheres colored in yellow, gray, and violet. **(A, B)** The side and top views of the complex cASIC1a/PcTx1 (PDB code 4FZ0). Toxins are shown using a red color. PcTx1 inserts its loop into the acidic pocket, simultaneously interacting with the finger and thumb domains of one channel subunit and the palm domain of adjacent subunit. **(C, D)** The side and top views of the complex cASIC1a/MitTx (PDB code 4NTW). Toxins’ α and β subunits are colored light orange and red, respectively. α-Subunit interacts with the wrist region and β1-β2 and β11-β12 linkers, whereas the β-subunit binds to the thumb and finger domains without penetration into the acidic pocket. **(E, F)** The model of the complex cASIC1a/mambalgin-1 is built based on the cryo-EM density map (Sun et al., 2018), crystal channel structure (PDB code 4FZ1), and crystal mambalgin-1 structure (PDB code 5DU1). Mambalgin-1 is shown using a red color. Loops of the polypeptide are numbered, and the mambalgin-1 form the contacts with the thumb domain by the first and second loops without penetration into the acidic pocket.

**Figure 11:**
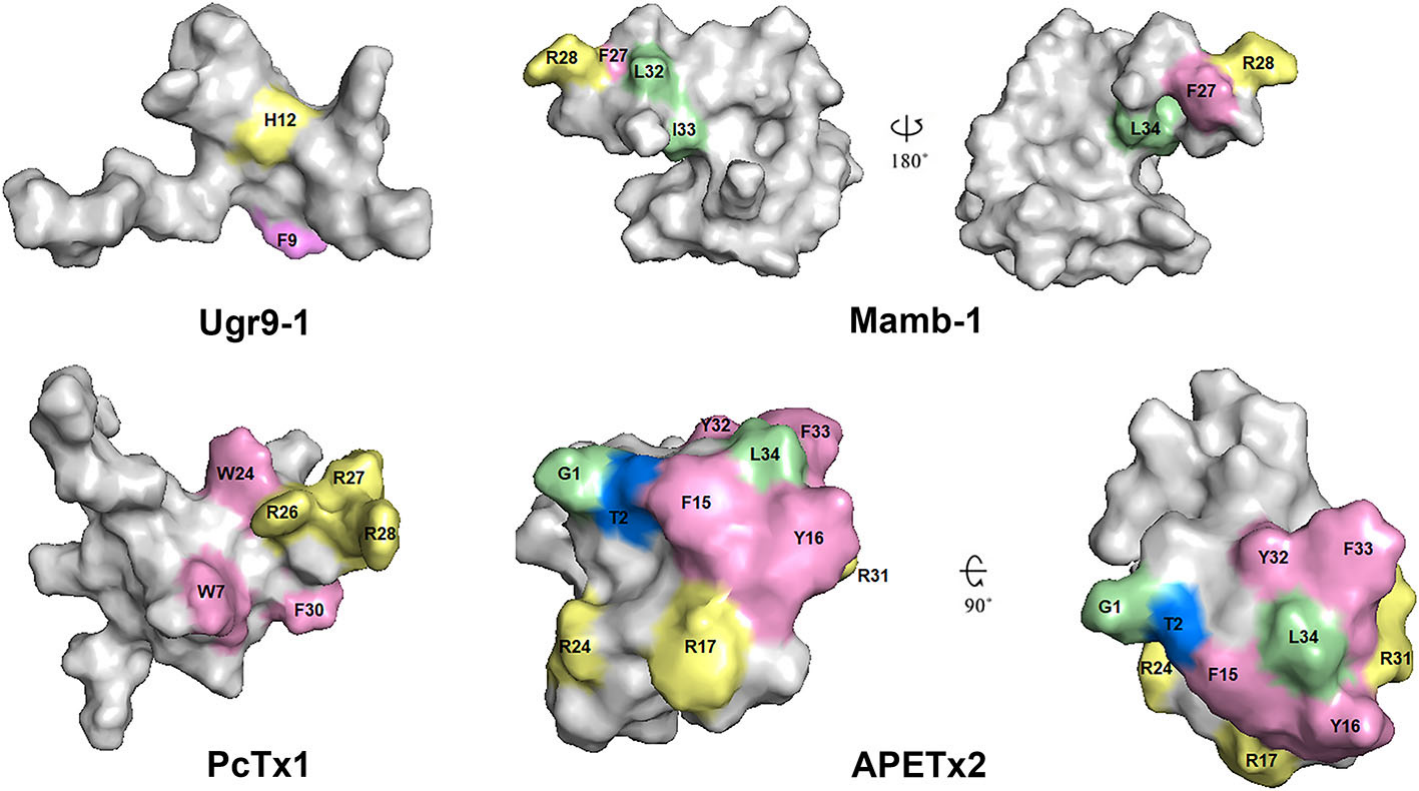
Spatial structure of toxins Ugr9-1 (PDB 2LZO), Mamb-1 (PDB 5DZ5), PcTx1 (PDB 2KNI), and APETx2 (PDB 2MUB). Marked residues play an important role in the activity of toxins on ASICs in accordance with scanning mutagenesis experiments. Basic, aromatic, and hydrophobic residues are indicated by light yellow, light lilac, and light green colors, respectively.

**Figure 12:**
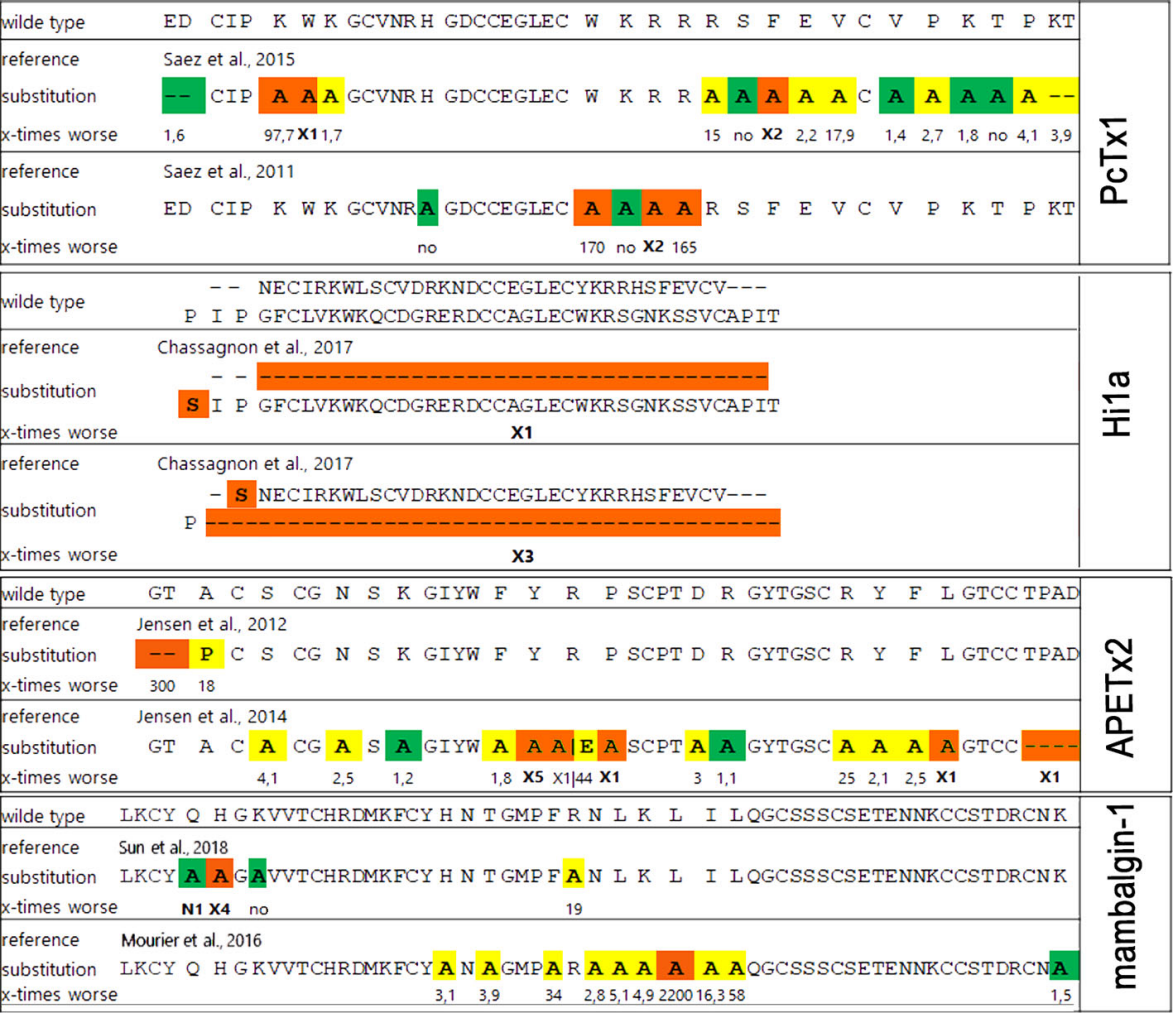
The influence of point mutation in polypeptide modulators on modulating activity to ASICs. Mutations without effect (green), medium affecting (yellow), and destroy the activity (orange) distributed in accordance with publications (references shown in picture). The decrease of mutants’ functional activity as part of the wild-type peptide activity for each substitution is presented below as a number except for: X1, loss of ability to inhibit ASICs; X2, loss of ability to inhibit rASIC1a at low concentration and gain of function as a positive modulator at high concentrations (>100 nM); X3, 2,600-fold decrease in inhibition of rASIC1a, and inhibition becomes fully reversible; X4, important decrease in inhibitory potency on cASIC1a; X5 more than 100-fold decrease in ability to inhibit rASIC3; N1, small increase in inhibitory potency on cASIC1a.

There are several structures of the cASIC1a complexes with different peptide ligands playing the role of agonists, inhibitors, and modulators (Jasti et al., 2007; Gonzales et al., 2009; Baconguis and Gouaux, 2012; Dawson et al., 2012; Baconguis et al., 2014; Sun et al., 2018; Yoder et al., 2018). In 2012, two separate groups from Switzerland and the USA published the structures of the cASIC1a complex with PcTx1 (Baconguis and Gouaux, 2012; Dawson et al., 2012). According to their studies, three molecules of PcTx1 bind to cASIC1a at the interfaces of two subunits about 45-50 Å above the membrane bilayer (**Figures 10A, B**). The binding is bimodal: by a hydrophobic patch and basic cluster of the toxin. The hydrophobic patch formed by Trp24, Trp7, Phe30, Val32, Val34, and Pro35 wraps around the thumb domain helix5, whereas the basic cluster formed by Arg26, Arg27, and Arg28 merges into the acidic pocket in contact with Asp350, Asp238, and Glu220, as well as with the palm domain of the adjacent subunit. Thus, PcTx1 simultaneously interacts with the palm, finger, and thumb domains of the channel and blocks their relative arrangement in the desensitized-like state (Baconguis and Gouaux, 2012; Dawson et al., 2012). Alanine-scanning mutagenesis of PcTx1 also showed that residues Trp7, Trp24, Arg26, Arg27, Arg28, and Phe30 are important for interaction with rat ASIC1a (Saez et al., 2015) (**Figures 11** and **12**). Due to the similarity of two Hi1a domains to PcTx1, it was obviously to study the effects of *N*- and *C*-terminal domains individually on ASIC1a (**Figure 12**). It was shown that, in contrast to native Hi1a, the *N*-terminal domain inhibits rASIC1a fully reversibly and much weaker (IC50 of 1.04 µM), whereas the *C*-terminal domain does not inhibit this channel subtype at all. *N*-terminal serine residues were vestiges of the fusion protein cleavage site (Chassagnon et al., 2017).

The crystal structure of the cASIC1a complex with MitTx revealed the open state of the channel (Baconguis et al., 2014). In this structure, each heterodimer of MitTx exclusively interacts with a single channel subunit forming the numerous contacts with the “wrist” region located near the membrane bilayer and with the β11-β12-linker by its α-subunit, as well as with the thumb domain located 60 Å above the membrane by the toxin’s β-subunit (**Figures 10C, D**). Thus, the toxin has a protruding contact area with the channel, although without direct interaction with the acidic pocket. Comparison of the cASIC1a structure in the desensitized state with the structure of the cASIC1a/MitTx complex points to the significantly increased intersubunit distance in the last case with overall expansion of the extracellular vestibule and symmetric open pore (Baconguis et al., 2014). At the same time, the ion selectivity filter does not undergo serious conformational transformations indicating the spatial independence of the channel gate and ion selectivity filter.

A significantly different mechanism of possible ASIC regulation based on the cryo-EM structure of the complex cASIC1a/mambalgin-1 was suggested in 2018 (Sun et al., 2018). In spite of a low resolution of the obtained structure (5.4 Å) without the ability to resolve the TM domain structure, a new mode of a peptide interaction with ASIC1a was revealed. It was shown that three molecules of mambalgin-1 bind to individual subunits of the channel, mainly forming contacts between the first and second loops of peptide and the thumb domain (**Figures 10E, F**). The residues from the first loop of mambalgin-1 (Gln5, His6, Lys8) form electrostatic contacts with the α4-helix of the thumb domain, whereas the second loop interacts with the α5-helix of the thumb domain by electrostatic (Arg28, Lys31) and hydrophobic (Met25, Pro26, Phe27, Leu30, Leu32) contacts. Complex formation with mambalgin-1 changes the conformation of the thumb domain, shifting it away from the threefold molecular axis. It leads to expansion of the acidic pocket and as a result could trap the channel into the closed state. Thus, mambalgin-1 acts as an allosteric negative modulator. This mechanism was confirmed by site-directed mutagenesis and electrophysiology studies of the mambalgin-1 and cASIC mutants (Sun et al., 2018), and it principally differs from the earlier proposed mechanism based on the computational modeling and site-directed mutagenesis, where mambalgin-1 penetrated the acidic pocket and interacted with the thumb domain of rat ASIC1a exclusively by the residues from the second loop (Phe27, Arg28, Leu32, Ile33, and Leu34) (Mourier et al., 2016; Sun et al., 2018) (**Figures 11** and **12**).

Since the ASIC1 subunit shares quite a low sequence identity with ASIC2, ASIC3, and ASIC4 subunits, further functional and structural studies of other members of the ASIC family still have a high challenge and require additional data for understanding of the mechanisms of ion binding, channel gating, and modulation using different ligands. Accordingly, several research groups are working on identification of pharmacophores for that inhibit ASIC3 channels. For example, the importance of *N*-terminus and other residues of APETx2 for an interaction with the channel was revealed (**Figures 11** and **12**). Specifically, Gly1, Thr2, Phe15, Tyr,16, Arg17, Arg24, Arg31, Tyr32, Phe33, and Leu34 are crucial for the inhibition or interaction with rat ASIC3 (Chagot et al., 2005; Jensen et al., 2012; Jensen et al., 2014). As mentioned above, APETx2 also inhibits the off-target hERG channels, and according to the mutagenesis study, surfaces responsible for the effect of the toxin on hERG and ASIC3 partially overlap (Jensen et al., 2014).

Another ligand of the ASIC3 channels is the peptide Ugr 9-1 that is cleaved from the common precursor protein during their maturation together with highly homologous peptides without ASIC3 activity. Mutagenesis of these inactive homologs showed that the key residues in Ugr 9-1 important for interaction with ASIC3 are Phe9 and His12 (**Figure 11**) (Osmakov et al., 2016). Therefore, the basic aromatic cluster of APETx2 and Ugr 9-1 important for interaction with ASIC3 resembles the situation with PcTx1, whose interaction with ASIC1a is also bimodal (Baconguis and Gouaux, 2012).

## Perspectives in Therapeutic Development

ASICs being the most sensitive to pH-change channels are at the forefront in the detection of normal and pathological stimuli in the neurons and other cells. Moderate changes in pH, together with a variety of other signals, could be an important signal in normal function (rapid local acidification in synapses during neuronal activity) and a sign of various pathological conditions (ischemia, inflammation, and cancer) (Rash and Rash, 2017). Peptides from venomous animals that are able to inhibit the activation of ASICs or decrease their expression could be considered useful hits for therapy of pathological states. The most studied channels that evidently take part in different normal and pathological processes are ASIC1a and ASIC3, while the significance of other ASICs is less understood, but some of them are also considered suitable for drug development.

Intensive studies of different ASIC modulators and knockout mice disclosed the role of these channels (at least ASIC1a and ASIC3) in detection of acidosis-mediated pain (Rash and Rash, 2017). Several pro-inflammatory endogenous mediators affect ASICs function and potentiate their response to acidification (arachidonic acid and RF-amide peptides (Askwith et al., 2000; Allen and Attwell, 2002), spermine, dynorphins, and histamine for ASIC1a (Babini et al., 2002; Sherwood et al., 2009; Nagaeva et al., 2016), lactate and serotonin for ASIC3 (Immke and McCleskey, 2001), and nocistatine and endogenous isoquinoline alkaloids (Osmakov et al., 2017; Osmakov et al., 2019a). Expression of ASIC1a in DRG neurons is elevated during inflammation and unregulated by inflammatory mediators (Voilley et al., 2001; Mamet et al., 2002). ASIC1a channels in the spinal dorsal horn neurons contribute to inflammatory hypersensitivity to pain (Duan et al., 2007). ASIC3 channels are considered to play a significant role in the perception of external stimuli in free nerve endings. They are present in a most part of muscle afferents and in a significant part of DRG neurons (Molliver et al., 2005; Ikeuchi et al., 2009). The administration of animal toxins can cause significant anti-inflammatory and analgesic effects by inhibiting ASICs’ function in PNS or/and CNS, as shown in the variety of animal models (Rash and Rash, 2017).

Peptide APETx2 was reported to produce a significant analgesic effect in several models of pain such as acid-induced pain, CFA-induced hyperalgesia, migraine-related pain, and postoperative pain (Karczewski et al., 2010; Deval et al., 2011; Callejo et al., 2015; Andreev et al., 2018; Lee et al., 2018; Holton et al., 2020). However, it should be taken into consideration that the ability of APETx2 to affect various subtypes of ASICs and several voltage-gated channels (as discussed above) limits connection between the effects of this peptide *in vivo* with ASIC3 function. Nevertheless, more selective to ASIC3, animal toxin Ugr 9-1 significantly reduces inflammatory and acid-induced pain at doses 0.01–1 mg/kg after intravenous or intramuscular administration (Osmakov et al., 2013; Andreev et al., 2018). Sevanol, the compound isolated from thyme, revealed high analgesic activity and was even more effective than peptide inhibitors of ASIC3, as was shown in a comparative study (Andreev et al., 2018). Sevanol efficacy could be a result of its ability to inhibit both components of the ASIC3 current and its additional ability to inhibit ASIC1a. Mambalgins are “multitarget” peptides from the venom of the black mamba and block heteromeric channels of ASIC1a and ASIC2a subunits in CNS neurons and ASIC1b-containing channels in peripheral sensory neurons. Mambalgins relieve the pain in several ways through inhibition of different subtypes of ASICs expressed both in central and peripheral neurons (Diochot et al., 2012; Diochot et al., 2016; Chang et al., 2019). Mambalgins intrathecal administration of ~340 pmol/mouse (~0.1 mg/kg dose) efficiently reduced different types of pain (e.g., acute heat pain, inflammatory hyperalgesia, formalin-induced pain) through an opioid-independent pathway involving ASIC1a and ASIC2a channels. Local injection of the same dose (~0.1 mg/kg) also produced a significant analgesic effect on acute heat pain and reversed carrageenan-induced inflammatory hyperalgesia *via* ASIC1b (Diochot et al., 2012). An intravenous administration 15-30 pmol/mouse (~0.005–0.01 mg/kg dose) of mambalgin-1 was reported to produce a significant analgesic effect in models of acid-induced mechanical hyperalgesia, thus confirming ASIC1b as a perspective pharmacological target on peripheral neurons (Chang et al., 2019).

ASICs play a significant role in a variety of processes in CNS, and the use of selective animal ligands helped to establish pharmacological perspectives of these channels’ modulators. In CNS, ASIC1a was involved in synaptic plasticity and learning (Wemmie et al., 2002; Du et al., 2014), fear conditioning, and fear behaviors (Wemmie et al., 2003; Wemmie et al., 2004; Coryell et al., 2008; Ziemann et al., 2009; Wemmie et al., 2013). Pharmacological inhibition of ASIC1a by PcTx1 or genetic deletion of ASIC1a strongly reduced the death of neurons and therefore the infarct volume in the model of ischemic stroke (McCarthy et al., 2015), whereas enhanced ASIC1a activity promoted the neuronal injury during ischemia in the animal model (Duan et al., 2011). PcTx1, injected in CNS, provides activation of the endogenous enkephalin pathway and naloxone-sensitive analgesia (Mazzuca et al., 2007). Also, PcTx1 exhibits neuroprotection activity of the *substantia nigra* in a model of Parkinson’s disease (Dwyer et al., 2009).

Low pH of extracellular environment and intracellular acidification accompanies tumor cell proliferation, metastasis, and tumor-related inflammation (Harguindey et al., 2017). Tumor cells express several pH-sensitive ion channels, including ASICs (Xu S. et al., 2016). Down-regulation of ASIC2a augments acidosis-mediated injury of C6 rat glioma cells (Liu et al., 2011). ASIC1a physically interacts with Ca^2+^/calmodulin-dependent protein kinase II (CaMKII) and integrin-β1 (Xu S. et al., 2016), which are important regulators of intracellular signaling and adhesion. Thus, ASICs can influence glioblastoma cell proliferation by different mechanisms. In addition to gliomas, ASICs are involved in regulation of lung (Wu et al., 2017), and other carcinomas growth, migration, as well as drug resistance (Gupta et al., 2016; Zhang et al., 2017). In line with it, benzamil and PcTx1 demonstrated antiproliferative activity against glioblastoma cells (Rooj et al., 2012), and mambalgin-2 against leukemia cells (Bychkov et al., 2020).

It is an interesting fact that targeted screening of chemical libraries led to only several drug seeds. Some of them, such as A317567 and A317567-10b, exhibited low selectivity to ASICs (Dubé et al., 2005; Kuduk et al., 2010), whereas others possessed limited *in vivo* activity such as NS383 (Munro et al., 2016) and PPC5650 (Nielsen et al., 2015). The most probable way to solve this problem is the modification of natural compounds from plants such as lindoldhamine (Osmakov et al., 2018; Osmakov et al., 2019c), gastrodin (Qiu et al., 2014), sevanol (Dubinnyi et al., 2012; Osmakov et al., 2015), and chlorogenic acid (Qu et al., 2014), as well as some prospective synthetic molecules recently discovered such as CHF5074 (Mango et al., 2014). These scaffolds could be a basis for the creation of the ASIC-oriented chemical libraries for a directed drug development. Noteworthy that the most of natural small molecules affecting ASICs possess activity on other cellular targets including voltage-gated sodium, potassium, and calcium channels with a same effectiveness. Measured *in vivo* effect for these compounds is a combination of different targets regulation that could be beneficial (synergic) in some cases but could be a reason for unpredictable side effects.

Modern medicine is waiting for selective and biologically stable ASIC modulators for the treatment of a great variety of pathological conditions. ASIC inhibitors could serve as effective analgesic and anti-inflammatory drugs, as well as neuroprotective substances for reducing damage from ischemic or traumatic injury of the brain. Moreover, the ASICs channel could play a significant role in the progression of some tumors and could be considered an anticancer target (Bychkov et al., 2020). Further study of the involvement of ASICs in various physiological processes using well-known and new toxins will allow researchers to find new medical problems associated with these channels’ functioning. We are convinced that natural compounds will always be extremely useful as tools for studies of animals’ main physiological processes and necessarily as remedies for healthcare.

## Author Contributions

DO, TK, YA, and EL performed bibliography analysis. DO, TK, EL, and SK prepared the figures. DO, TK, YA, EL, and SK wrote the manuscript. SK and YA critically reviewed the manuscript.

## Funding

The work was supported by the Russian Science Foundation (project # 19-74-20163).

## Conflict of Interest

The authors declare that the research was conducted in the absence of any commercial or financial relationships that could be construed as a potential conflict of interest.

## Abbreviations

5-CQA, 5-caffeoylquinic acid; ASICs, acid-sensing ion channels; α-DTx, α-dendrotoxin; BBH, boundless β-hairpin; CNS, central nervous system; CNFs, cono-RFamides; DAU, daurisoline; DRG, dorsal root ganglion; EC_50_, half-maximal effective concentration; ECDs, extracellular domains; EGCG, (-)-Epigallocatechin gallate; IC_50_, half-maximal inhibitory concentration; ICK, inhibitor cystine knot; LIN, lindoldhamine; NEO, neomycin; nH, Hill coefficient; NMR, nuclear magnetic resonance; pH_50_, half-activation pH; PcTx1, psalmotoxin; PNS, peripheral nervous system; SSD, steady-state desensitization; ST, streptomycin; TM, transmembrane; TRPV1, transient receptor potential V1.
